# A case report of ruptured amoebic liver abscess causing cardiac tamponade and requiring pericardial window

**DOI:** 10.1093/ehjcr/ytaa182

**Published:** 2020-08-30

**Authors:** Cliojis Francis, Swati Soni, Anunay Gupta, Sourabh Agstam

**Affiliations:** Department of Cardiology, VMMC and Safdarjung Hospital, New Delhi, India; Postgraduate Institute of Medical Education and Research (PGIMER), Chandigarh, India; Department of Cardiology, VMMC and Safdarjung Hospital, New Delhi, India; Department of Cardiology, VMMC and Safdarjung Hospital, New Delhi, India; Postgraduate Institute of Medical Education and Research (PGIMER), Chandigarh, India

**Keywords:** Amoebic liver abscess, Cardiac tamponade, Pericardiocentesis, Pleural effusion, Pericardial window, Case report

## Abstract

**Background:**

Amoebiasis is a prevalent infection in the tropics. Amoebic liver abscess is the most common extraintestinal manifestation. Cardiac tamponade is an uncommon complication of amoebic liver abscess that may need urgent pericardiocentesis.

**Case summary:**

A 25-year-old man presented with abdominal pain and fever for 1 month. Abdominal ultrasound revealed a 4.7 × 4.7 cm abscess in the left lobe of the liver. Percutaneous pigtail drainage was performed to evacuate the abscess. After 2 days, the patient developed signs of cardiac tamponade and bilateral pleural effusion, requiring urgent pericardiocentesis and chest drain insertion. Persistent posterior collection of thick abscess in pericardium needed pericardial window for complete drainage. The patient recovered completely after pericardial window. There was no evidence of chronic constrictive pericarditis after 1 year of follow-up.

**Discussion:**

A rare complication of the amoebic liver abscess was observed in this young adult who developed cardiac tamponade, requiring an urgent pericardiocentesis, and later requiring pericardial window. Management includes amoebicidal and luminicidal drugs for complete eradication of *Entamoeba histolytica*.

**Learning points**

- An amoebic liver abscess can rupture into the pericardium and pleural cavity even after drainage.
- The early diagnosis and strong index of suspicion of tamponade are important for treatment and prevention of mortality.
- The liver abscess should always be managed under observation in the hospital until the complete resolution of the abscess.
- Management of complicated amoebic liver abscess needs multispecialty involvement.

## Introduction

Amoebiasis is a prevalent infection in the tropics. It presents with fever, bloody diarrhoea and can spread to other organs either by direct extension from the intestine or by blood–borne transmission if not treated promptly. The commonest form of the metastatic disease affects the liver and presents as amoebic hepatitis or amoebic abscess. Rupture of the amoebic liver abscess into the pericardium is a rare complication with high mortality causing cardiac tamponade, as a result of rupture of the left lobe liver abscess or direct extension of the right-sided pleural amoebiasis.^1^^,^^2^ Herein, we present a case of an adult with left lobe amoebic liver abscess that, ruptured into the pericardium and pleural cavity complicated by a posterior collection of abscess in the pericardiumrequiring a pericardial window to prevent reaccumulation.

## Timeline

**Table:** 

Presentation	The patient had a history of abdominal pain and fever for 1 month and received empirical oral ofloxacin 500 mg for 1 week without any relief.An amoebic liver abscess in left lobe of size 4.7 × 4.7cm was diagnosed on abdominal ultrasonography. Around 100 mL of brown colour pus (anchovy sauce) was drained. He was started on intravenous metronidazole 400 mg thrice daily.
Days 3–4	Patient had shortness of breath associated with hypotension. A two-dimensional transthoracic echocardiogram showed massive purulent pericardial effusion causing cardiac tamponade. Urgent pericardiocentesis was done and 700 mL of brown colour pus drained. Chest X-ray revealed bilateral moderate pleural effusion with empyema. Pigtail was inserted bilaterally and 500 mL of serosanguinous fluid was drained.
Days 5–10	Fever persisted; cultures from the drain grew *Acinetobacter baumannii*. Patient was started on injection colistin.
Days 11–12	Non-contrast computed tomography chest revealed 5.7 cm thick pus collection posteriorly in pericardial cavity. Pericardial window was created and his symptoms convalescence. Pericardial and pleural drains were removed; the patient discharged with no post-operative complications.
1, 6, and 12 months	He started his job after 1 month. Regular follow-up at 6 months and at 1 year was with in normal limits.

## Case presentation

A 25-year-old man, teetotaller, with no previous comorbidities presented to the emergency department with history of abdominal pain and fever for 1 month. He had received empirical antibiotics and proton pump inhibitors without any symptomatic relief. There was no history of similar complaints in the past and the family history was non-contributory. On examination, the patient was malnourished with body mass index of 16.9 kg/m^2^. He was febrile, blood pressure of 118/62 mmHg, pulse rate of 112/min, and respiratory rate of 14/min. Abdominal examination revealed mild epigastric tenderness without any rigidity and bowel sounds were intact.

Results and reference values, according to age and sex, of the most relevant laboratorial tests performed in our patient at presentation

**Table:** 

Laboratorial examination	Results	Reference value
Haemoglobin	9.1 g/dL	13.5–17.5 g/dL
Creatinine	0.8 mg/dL	0.7–1.2 mg/dL
Total leucocyte count	21 700/µL	4.0–11.0 × 10^3^/µL
Total bilirubin	6.78 mg/dL	0.0–1.4 mg/dL
Conjugated bilirubin	3.66 mg/dL	0.0–0.3 mg/dL
SGOT	1393 µ/L	5–40 µ/L
SGPT	2117 µ/L	7–56 µ/L
Alkaline phosphatase	239 µ/L	20–140 µ/L
HbsAg	Non-reactive	
HIV 1&2	Non-reactive	
Anti HCV	Non-reactive	

Anti HCV, antibody to Hepatitis C virus; HbsAg, Hepatitis B surface antigen; HIV, Human Immunodeficiency virus; SGOT, serum glutamic oxaloacetic transaminase; SGPT, serum glutamic pyruvic transaminase.

On abdominal ultrasonography, a liver abscess of 4.7 × 4.7 cm in the left lobe of the liver was noted and a percutaneous pigtail catheter drainage under local anaesthesia aspirated 100 mL of anchovy sauce-like pus. Although no amoeba was cultivated from the pus, the polymerase chain reaction from the pus was positive for *Entamoeba histolytica*. Therefore, he was started on intravenous metronidazole 400 mg thrice daily.

**Figure 1:**
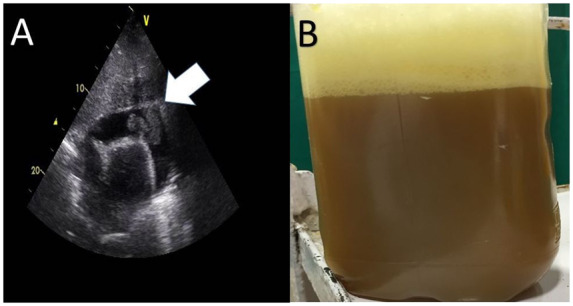
(*A*) A transthoracic echocardiogram four-chamber view from the subxiphoid approach showed massive pus collection in the pericardial cavity leading to cardiac tamponade. (*B*) A 700 mL brown colour pus-like material (Anchovy sauce) was aspirated from the pericardium.

After 2 days, he developed shortness of breath associated with severe chest pain. He had tachypnoea, muffled heart sounds, and jugular venous distension associated with hypotension. Transthoracic echocardiography revealed cardiac tamponade with massive purulent pericardial effusion (*Figure 1*, *Video 1*).

**Figure 2:**
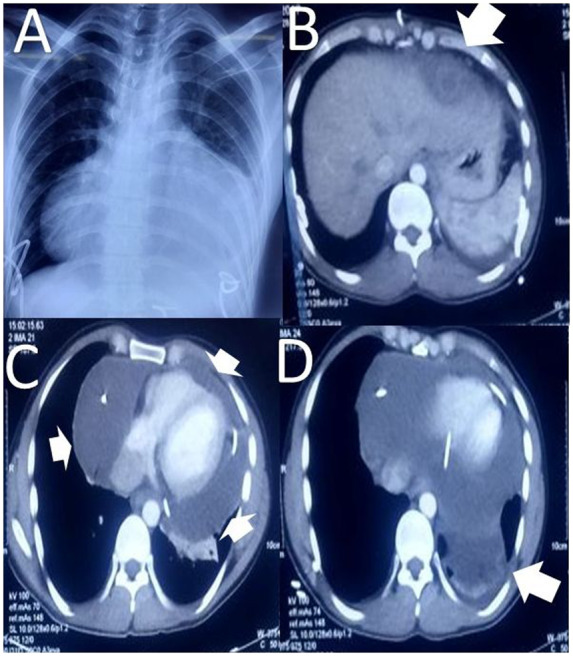
(*A*) Chest X-ray of a patient with amoebic liver abscess showing cardiomegaly with left side pleural effusion. (*B*) Non-contrast computed tomography abdomen demonstrated minimal residual collection in segment II of liver (white arrow). (*C*) Thick pus collection in pericardium with minimum effusion anteriorly on non-contrast computed tomography (white arrows). (*D*) Empyema in the left pleural cavity (white arrow).

He underwent emergency subxiphoid percutaneous pigtail catheter drainage under local anaesthesia leading to symptomatic relief; however, he continued to have chest pain and chest X-ray showed bilateral empyema and percutaneous pigtails under local anaesthesia were inserted into both pleural cavities draining around 200–300 mL serosanguinous fluid. His fever persisted and there was 100 mL of daily drainage from pericardial drain. Cultures from the drain grew *Acinetobacter baumanii*, and the patient was started on intravenous colistin along with intravenous metronidazole. An abdominal ultrasonography reassessment revealed no residual abscess in the liver, so the liver catheter was removed. Still, there were residual collections in the pleural and pericardial cavities. A repeat transthoracic echocardiography revealed minimal fluid anterior to the heart with 10 mm pericardial effusion lateral and posterior to the left ventricle. Pigtail was repositioned and 20–30 mL of pus aspirated. Non-contrast computed tomography abdomen and chest showed a 5.7 cm thick purulent pericardial collection on the lateral side of the right ventricle and bilateral empyema in lung (*Figure 2*).

A decision for surgical drainage was considered and a pleura-pericardial window was performed under general anaesthesia draining 800 mL of pus. Following post-operative Day 1, he improved symptomatically and his fever alleviated. The rest of the post-operative days were uneventful and the pleural and pericardial drains were removed after 7 days. Following surgery, he recovered well and the fever subsided. The patient was discharged after 10 days of surgery. He started his job after 1 month of discharge and is doing well after 1 year of follow-up with no recurrence of the disease.

## Discussion

Amoebiasis affects approximately 12% of the population of the world. It is a benign condition with occasionally having no clinical symptoms while the intestinal invasive disease manifests with abdominal cramping, watery diarrhoea, and weight loss.^3^ The strain virulence, environment, host’s genetic susceptibility, immune status, age, and gender determine the prognosis of the illness.^3^ The pathogenesis of the invasive disease is due to adherence and lysis of the colonic epithelium by the trophozoites and haematological spread through the portal vein system to distant sites such as peritoneum, liver, lung, or brain.^4^

Amoebic liver abscess is one of the most common complication affecting around 3–9% of patients leading to significant morbidity and mortality.^4^^,^^5^ It is more prevalent amongst males than females and can develop at any age, although mostly affecting between 18 and 50 years of age.^6^^,^^7^ Interestingly, the amoebic abscess is a misnomer as the pus contains necrotic liver tissue with a thin-walled granulation. The liver abscess are frequently localized in the right lobes and are usually single, while the incidence of extraintestinal involvement is higher when the left lobe of the liver is involved.^8^

Metronidazole remains the drug of choice for amoebic liver abscess. The use of amoebicides in treatment has significantly decreased the mortality and morbidity due to the disease. After the patient has been treated with metronidazole for the liver abscess, a luminal agent such as paromomycin and diloxanide furoate should be used for the treatment of asymptomatic colonization state and prevent relapse of infection.^8^ Therapeutic aspiration by imaging-guided percutaneous interventions (needle aspirations, pigtail catheters) of liver abscess should be contemplated in a patient with a high risk of rupture that includes large abscess (cavity diameter > 5 cm), left lobe abscess due to risk of rupture into the pericardial sac, and liver abscess resistant to 5–7 days of medical management.^9^

## Lead author biography

**Figure:**
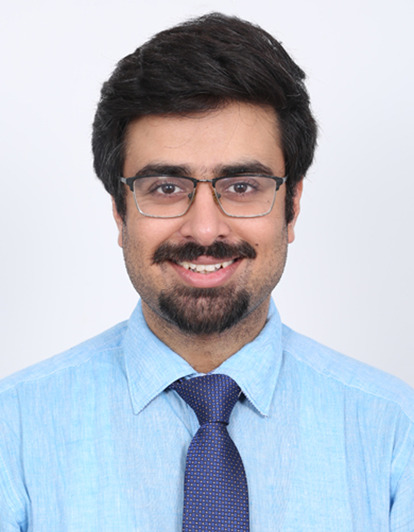


Sourabh Agstam is a postgraduate in Medicine from All India Institute of Medical Sciences (AIIMS, New Delhi), had residency training in cardiology at PGIMER, Chandigarh. Currently, he is working as Assistant Professor in Department of Cardiology in VMMC and Safdarjung Hospital, New Delhi. His fields of interest are in cardiac physiology, cardiac infections, and electrophysiology.

## Supplementary material

Supplementary material is available at *European Heart Journal - Case Reports* online.

**Slide sets:** A fully edited slide set detailing this case and suitable for local presentation is available online as Supplementary data.

**Consent:** The author/s confirm that written consent for submission and publication of this case report including image(s) and associated text has been obtained from the patient in line with COPE guidance.

**Conflict of interest**: none declared.

## Supplementary Material

ytaa182_Supplementary_Data
