# Supplementary material for: A case report of ruptured amoebic liver abscess causing cardiac tamponade and requiring pericardial window
Source: Eur Heart J Case Rep. 2020 Aug 30;4(5):1–4. doi: 10.1093/ehjcr/ytaa182 (PMC7780479; doi:10.1093/ehjcr/ytaa182)
Supplement: ytaa182_Supplementary_Data [file ytaa182_supplementary_data.zip › ytaa182_Supplementary_Data/slide set for case study 25-04-2020.pptx]

## Slide 1
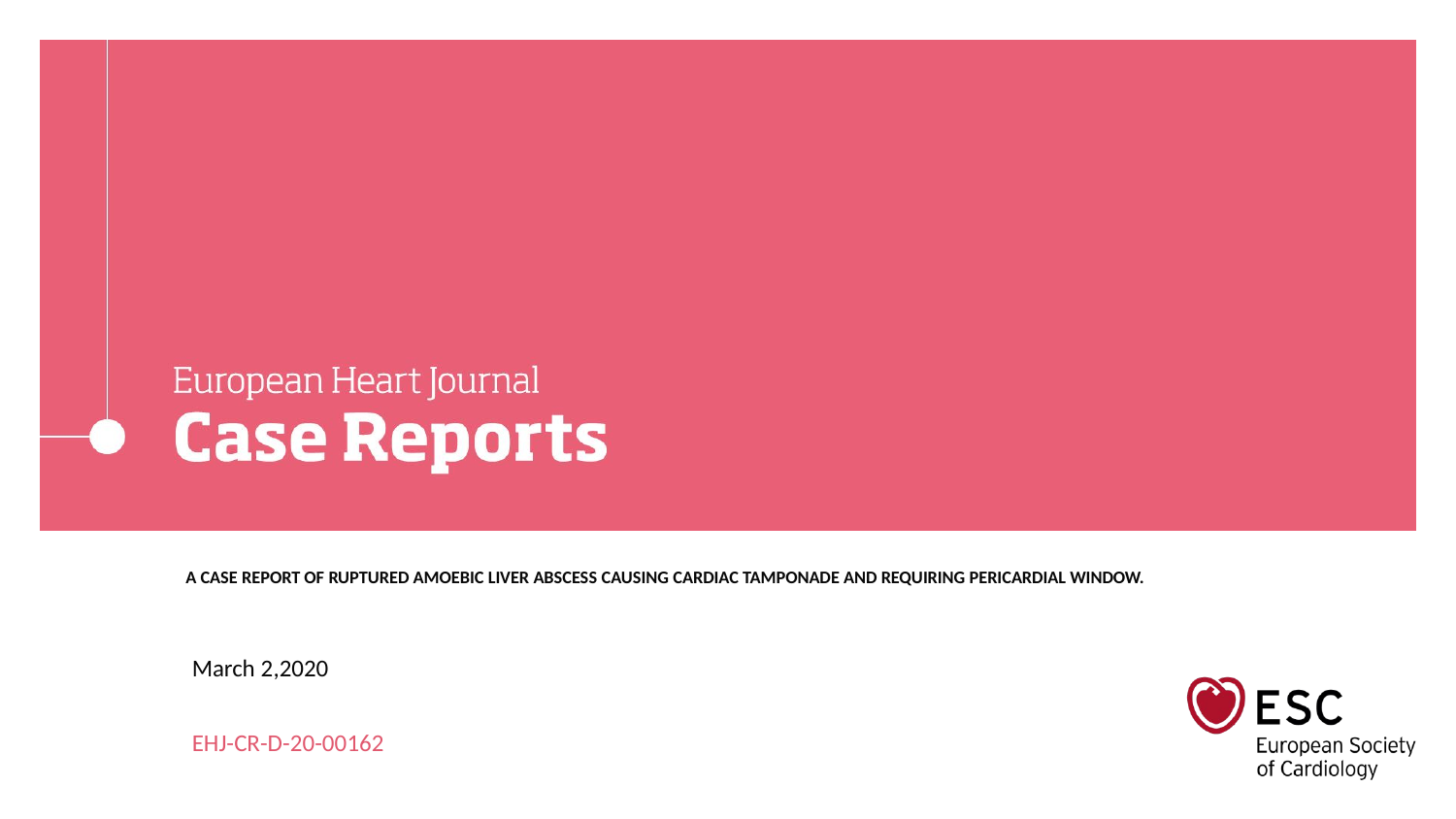

# A CASE REPORT OF RUPTURED AMOEBIC LIVER ABSCESS CAUSING CARDIAC TAMPONADE AND REQUIRING PERICARDIAL WINDOW.
March 2,2020
EHJ-CR-D-20-00162

## Slide 2
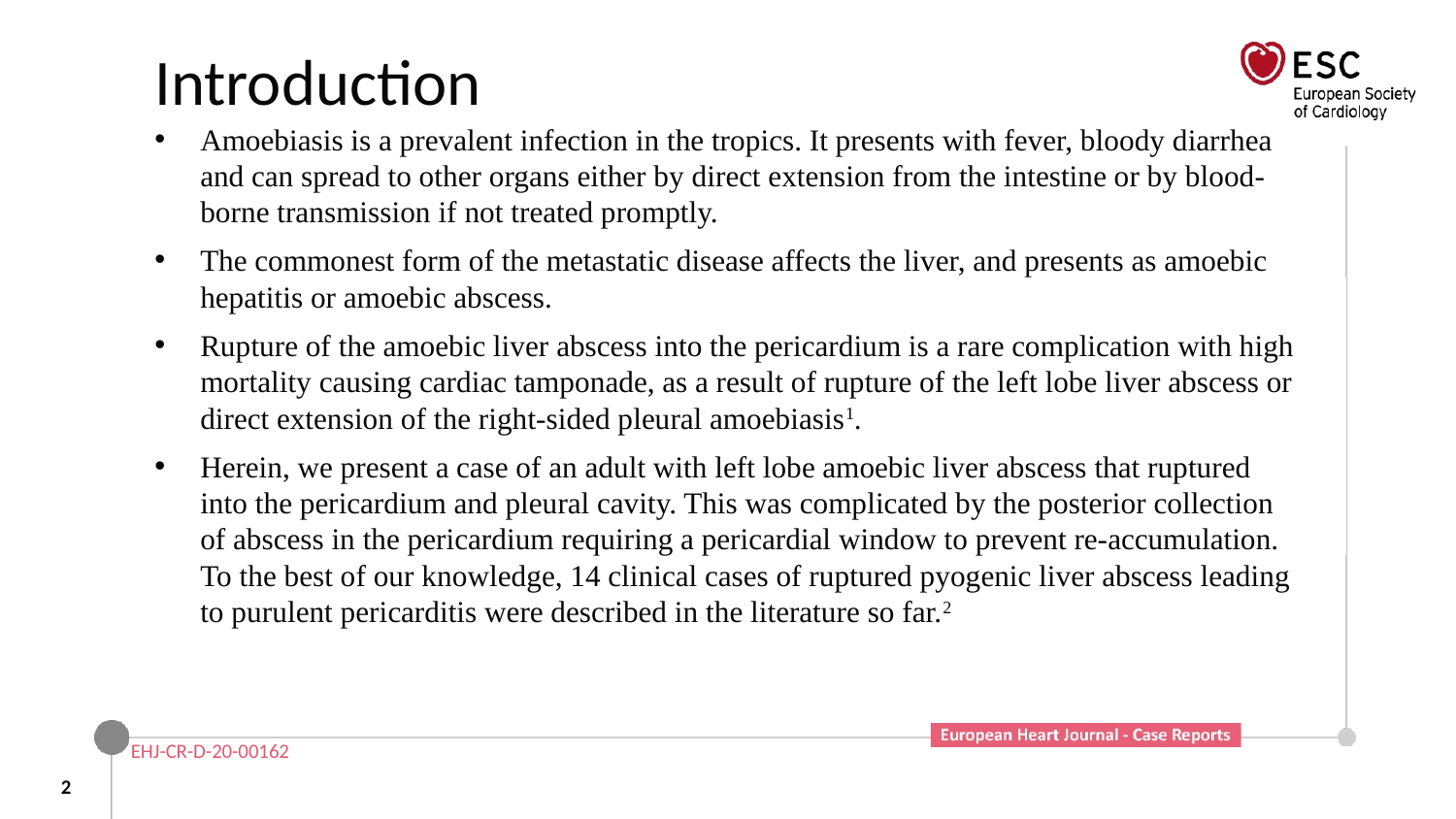

# Introduction
Amoebiasis is a prevalent infection in the tropics. It presents with fever, bloody diarrhea and can spread to other organs either by direct extension from the intestine or by blood-borne transmission if not treated promptly.
The commonest form of the metastatic disease affects the liver, and presents as amoebic hepatitis or amoebic abscess.
Rupture of the amoebic liver abscess into the pericardium is a rare complication with high mortality causing cardiac tamponade, as a result of rupture of the left lobe liver abscess or direct extension of the right-sided pleural amoebiasis1.
Herein, we present a case of an adult with left lobe amoebic liver abscess that ruptured into the pericardium and pleural cavity. This was complicated by the posterior collection of abscess in the pericardium requiring a pericardial window to prevent re-accumulation. To the best of our knowledge, 14 clinical cases of ruptured pyogenic liver abscess leading to purulent pericarditis were described in the literature so far.2
EHJ-CR-D-20-00162
2

## Slide 3
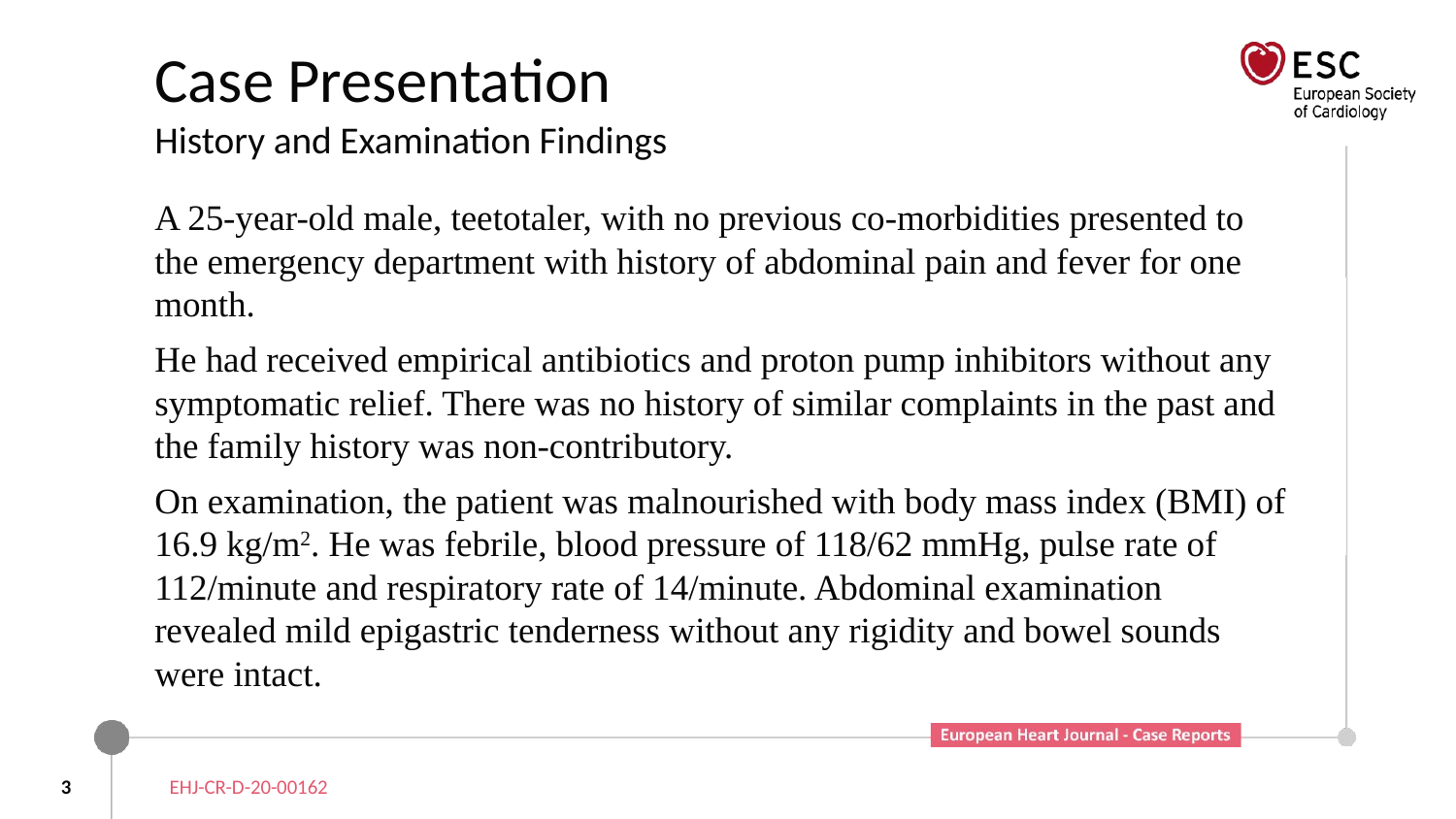

# Case Presentation History and Examination Findings
A 25-year-old male, teetotaler, with no previous co-morbidities presented to the emergency department with history of abdominal pain and fever for one month.
He had received empirical antibiotics and proton pump inhibitors without any symptomatic relief. There was no history of similar complaints in the past and the family history was non-contributory.
On examination, the patient was malnourished with body mass index (BMI) of 16.9 kg/m2. He was febrile, blood pressure of 118/62 mmHg, pulse rate of 112/minute and respiratory rate of 14/minute. Abdominal examination revealed mild epigastric tenderness without any rigidity and bowel sounds were intact.
3
EHJ-CR-D-20-00162

## Slide 4
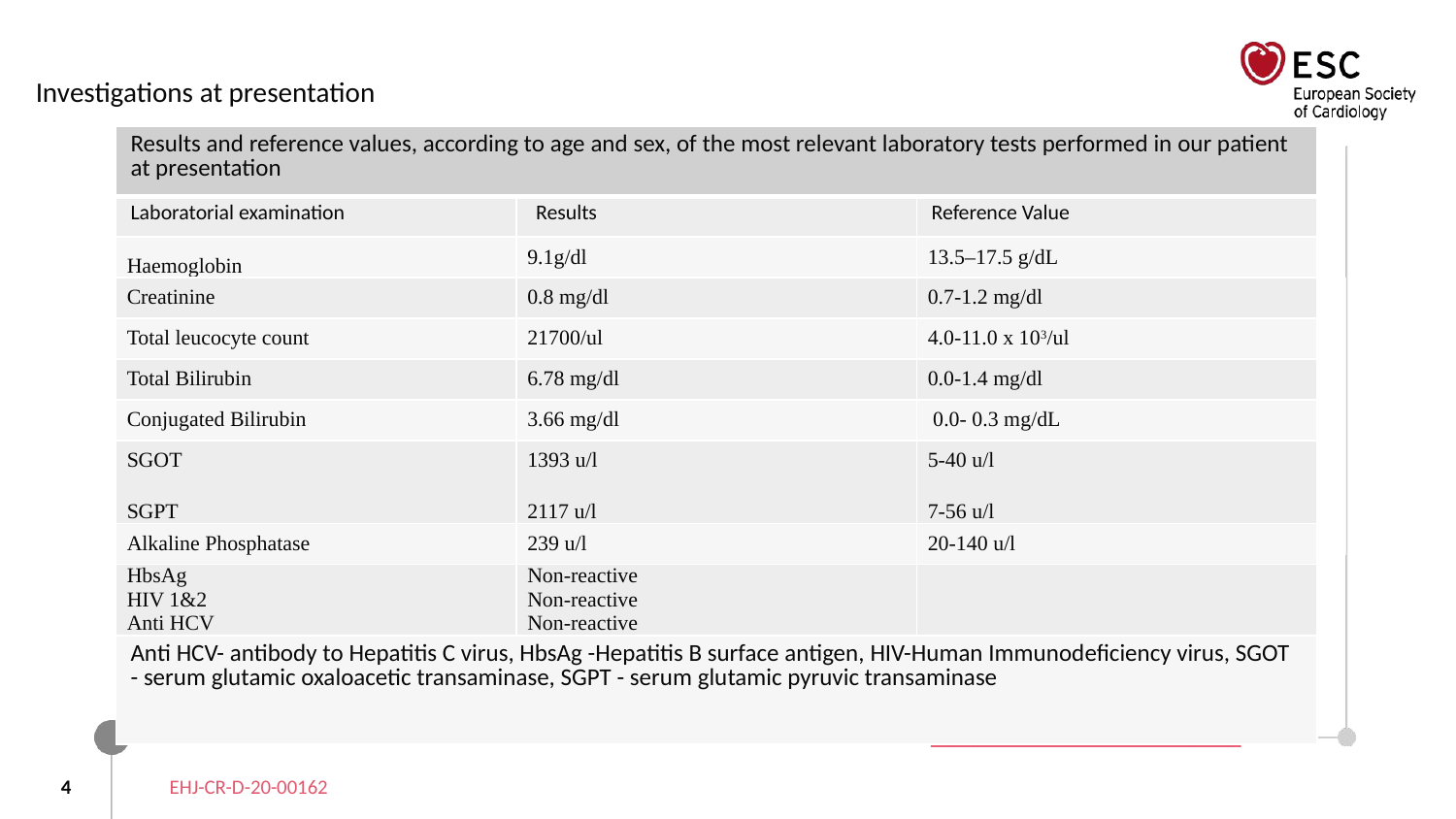

# Investigations at presentation
| Results and reference values, according to age and sex, of the most relevant laboratory tests performed in our patient at presentation | | |
| --- | --- | --- |
| Laboratorial examination | Results | Reference Value |
| Haemoglobin | 9.1g/dl | 13.5–17.5 g/dL |
| Creatinine | 0.8 mg/dl | 0.7-1.2 mg/dl |
| Total leucocyte count | 21700/ul | 4.0-11.0 x 103/ul |
| Total Bilirubin | 6.78 mg/dl | 0.0-1.4 mg/dl |
| Conjugated Bilirubin | 3.66 mg/dl | 0.0- 0.3 mg/dL |
| SGOT SGPT | 1393 u/l 2117 u/l | 5-40 u/l 7-56 u/l |
| Alkaline Phosphatase | 239 u/l | 20-140 u/l |
| HbsAg HIV 1&2 Anti HCV | Non-reactive Non-reactive Non-reactive | |
| Anti HCV- antibody to Hepatitis C virus, HbsAg -Hepatitis B surface antigen, HIV-Human Immunodeficiency virus, SGOT - serum glutamic oxaloacetic transaminase, SGPT - serum glutamic pyruvic transaminase | | |
4
EHJ-CR-D-20-00162

## Slide 5
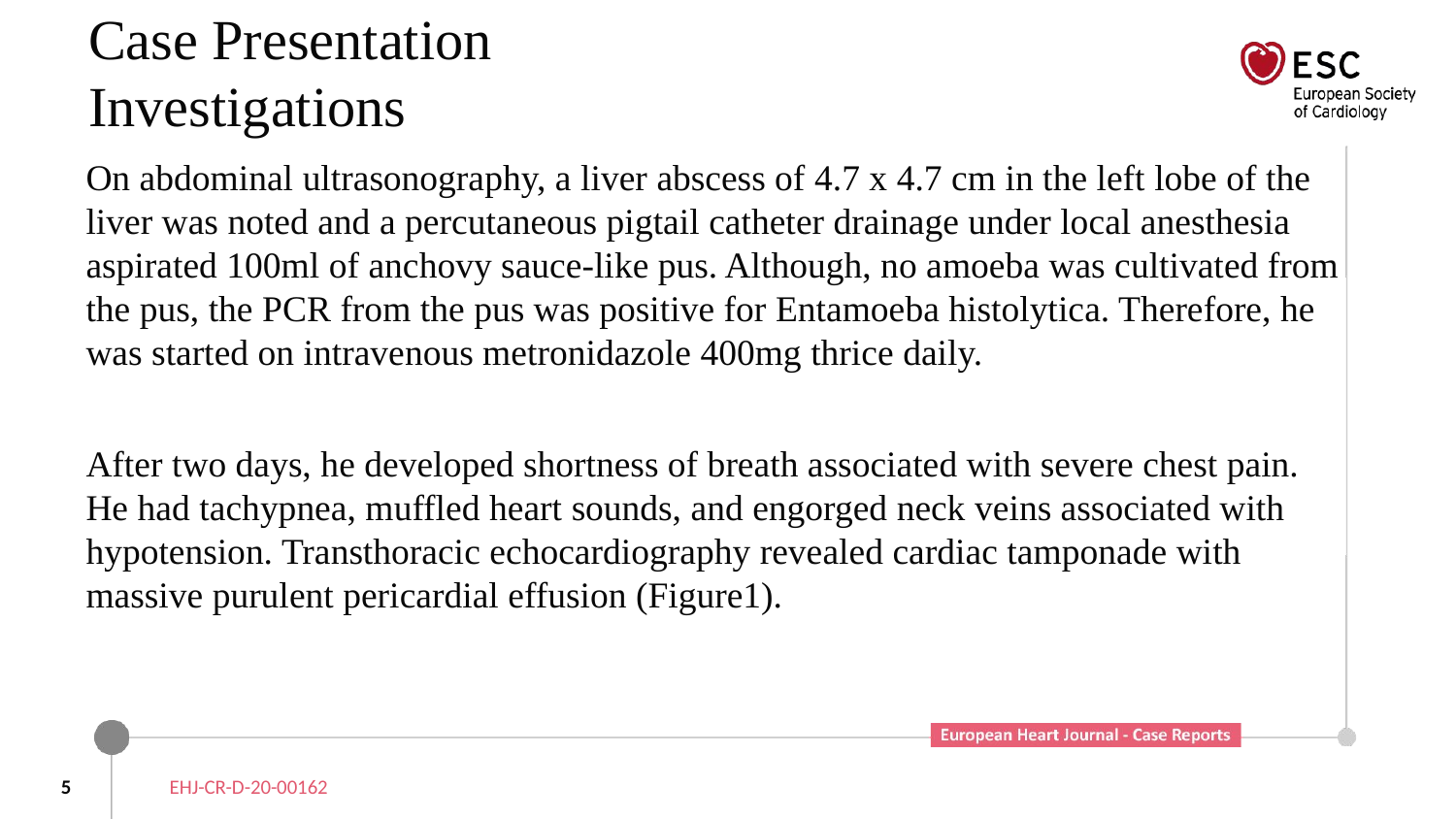

# Case Presentation Investigations
On abdominal ultrasonography, a liver abscess of 4.7 x 4.7 cm in the left lobe of the liver was noted and a percutaneous pigtail catheter drainage under local anesthesia aspirated 100ml of anchovy sauce-like pus. Although, no amoeba was cultivated from the pus, the PCR from the pus was positive for Entamoeba histolytica. Therefore, he was started on intravenous metronidazole 400mg thrice daily.
After two days, he developed shortness of breath associated with severe chest pain. He had tachypnea, muffled heart sounds, and engorged neck veins associated with hypotension. Transthoracic echocardiography revealed cardiac tamponade with massive purulent pericardial effusion (Figure1).
5
EHJ-CR-D-20-00162

## Slide 6
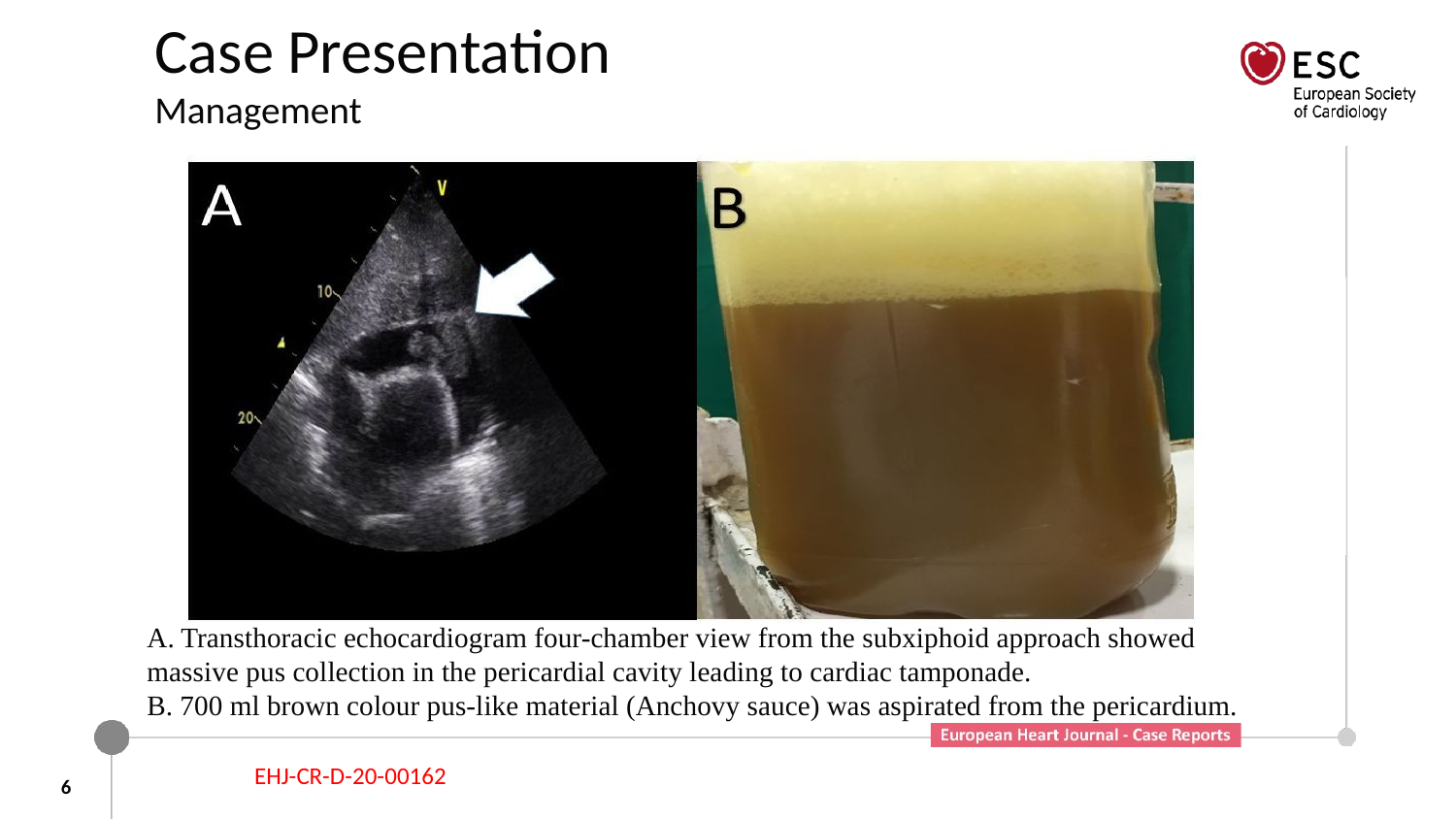

# Case Presentation Management
A. Transthoracic echocardiogram four-chamber view from the subxiphoid approach showed massive pus collection in the pericardial cavity leading to cardiac tamponade.
B. 700 ml brown colour pus-like material (Anchovy sauce) was aspirated from the pericardium.
 EHJ-CR-D-20-00162
6

## Slide 7
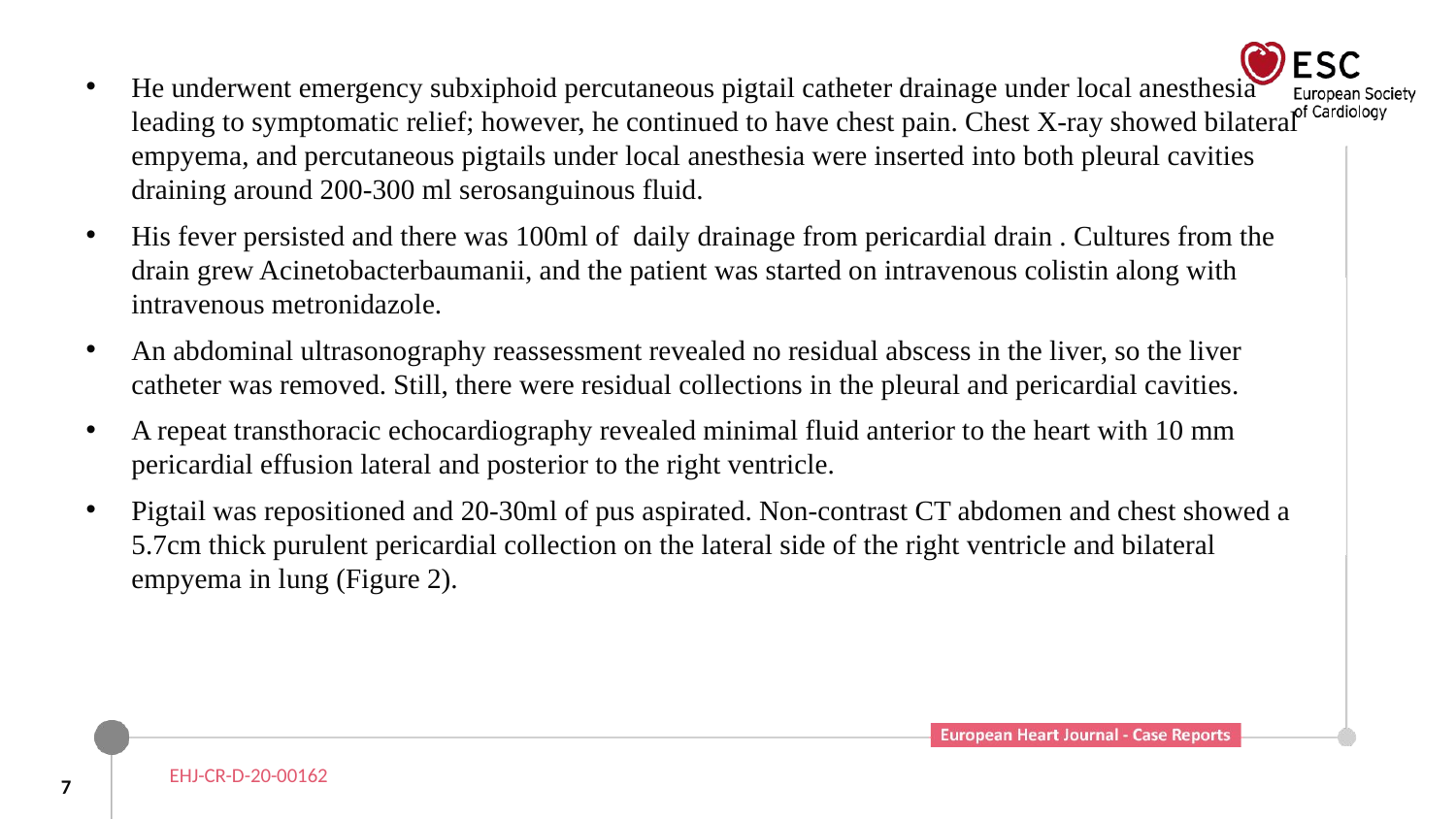

He underwent emergency subxiphoid percutaneous pigtail catheter drainage under local anesthesia leading to symptomatic relief; however, he continued to have chest pain. Chest X-ray showed bilateral empyema, and percutaneous pigtails under local anesthesia were inserted into both pleural cavities draining around 200-300 ml serosanguinous fluid.
His fever persisted and there was 100ml of daily drainage from pericardial drain . Cultures from the drain grew Acinetobacterbaumanii, and the patient was started on intravenous colistin along with intravenous metronidazole.
An abdominal ultrasonography reassessment revealed no residual abscess in the liver, so the liver catheter was removed. Still, there were residual collections in the pleural and pericardial cavities.
A repeat transthoracic echocardiography revealed minimal fluid anterior to the heart with 10 mm pericardial effusion lateral and posterior to the right ventricle.
Pigtail was repositioned and 20-30ml of pus aspirated. Non-contrast CT abdomen and chest showed a 5.7cm thick purulent pericardial collection on the lateral side of the right ventricle and bilateral empyema in lung (Figure 2).
7
EHJ-CR-D-20-00162

## Slide 8
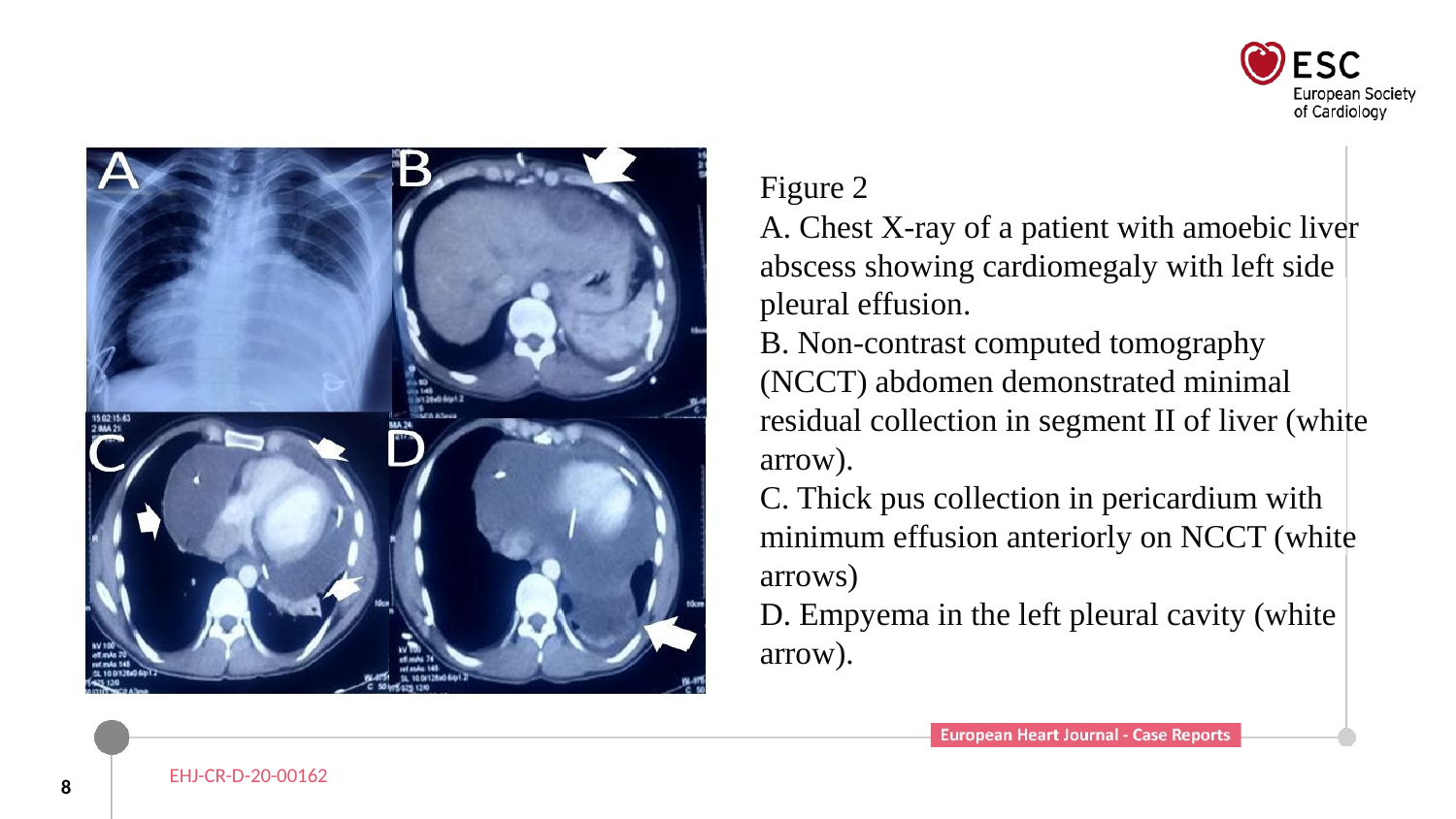

Figure 2
A. Chest X-ray of a patient with amoebic liver abscess showing cardiomegaly with left side pleural effusion.
B. Non-contrast computed tomography (NCCT) abdomen demonstrated minimal residual collection in segment II of liver (white arrow).
C. Thick pus collection in pericardium with minimum effusion anteriorly on NCCT (white arrows)
D. Empyema in the left pleural cavity (white arrow).
8
EHJ-CR-D-20-00162

## Slide 9
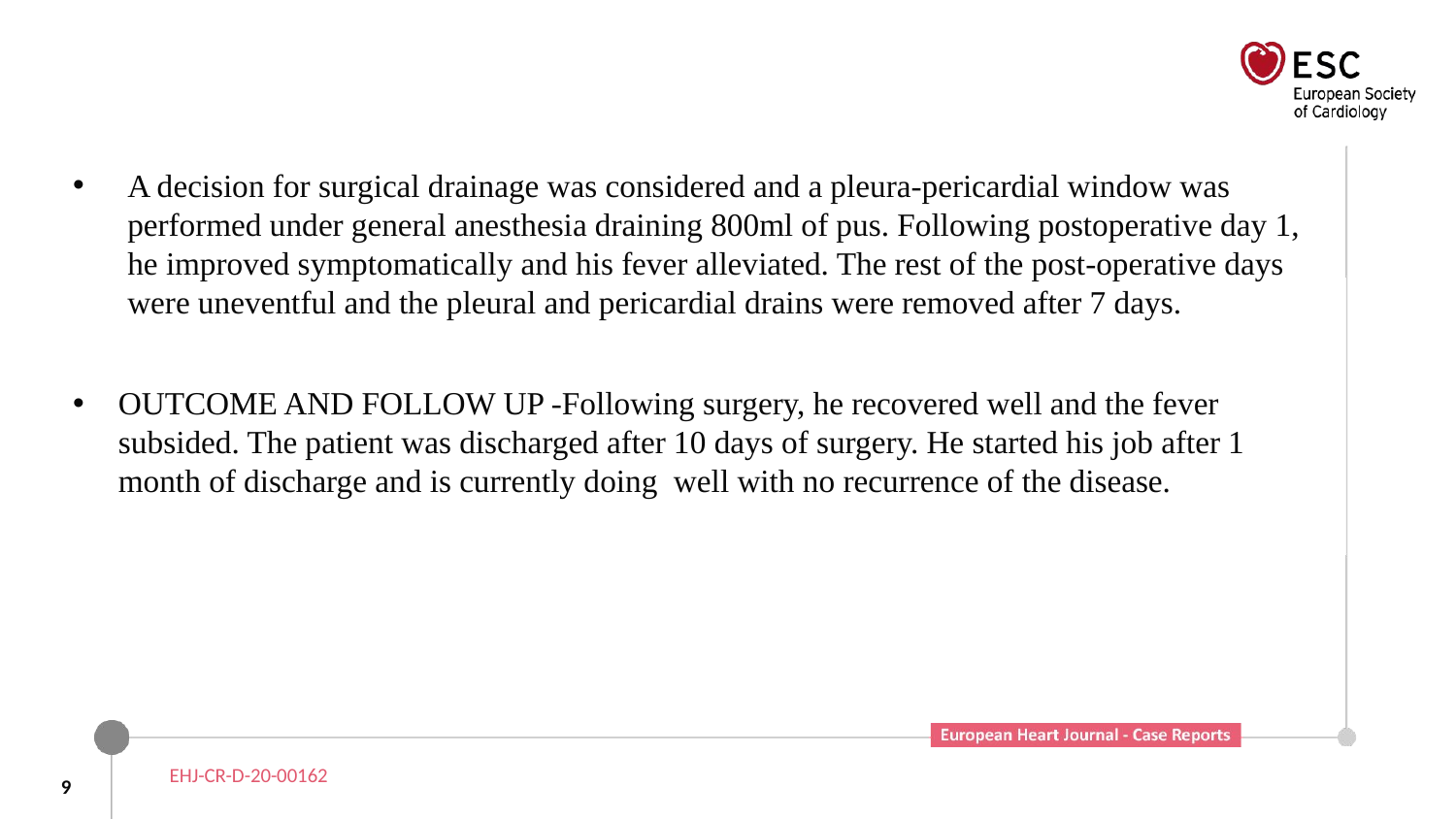

A decision for surgical drainage was considered and a pleura-pericardial window was performed under general anesthesia draining 800ml of pus. Following postoperative day 1, he improved symptomatically and his fever alleviated. The rest of the post-operative days were uneventful and the pleural and pericardial drains were removed after 7 days.
OUTCOME AND FOLLOW UP -Following surgery, he recovered well and the fever subsided. The patient was discharged after 10 days of surgery. He started his job after 1 month of discharge and is currently doing well with no recurrence of the disease.
9
EHJ-CR-D-20-00162

## Slide 10
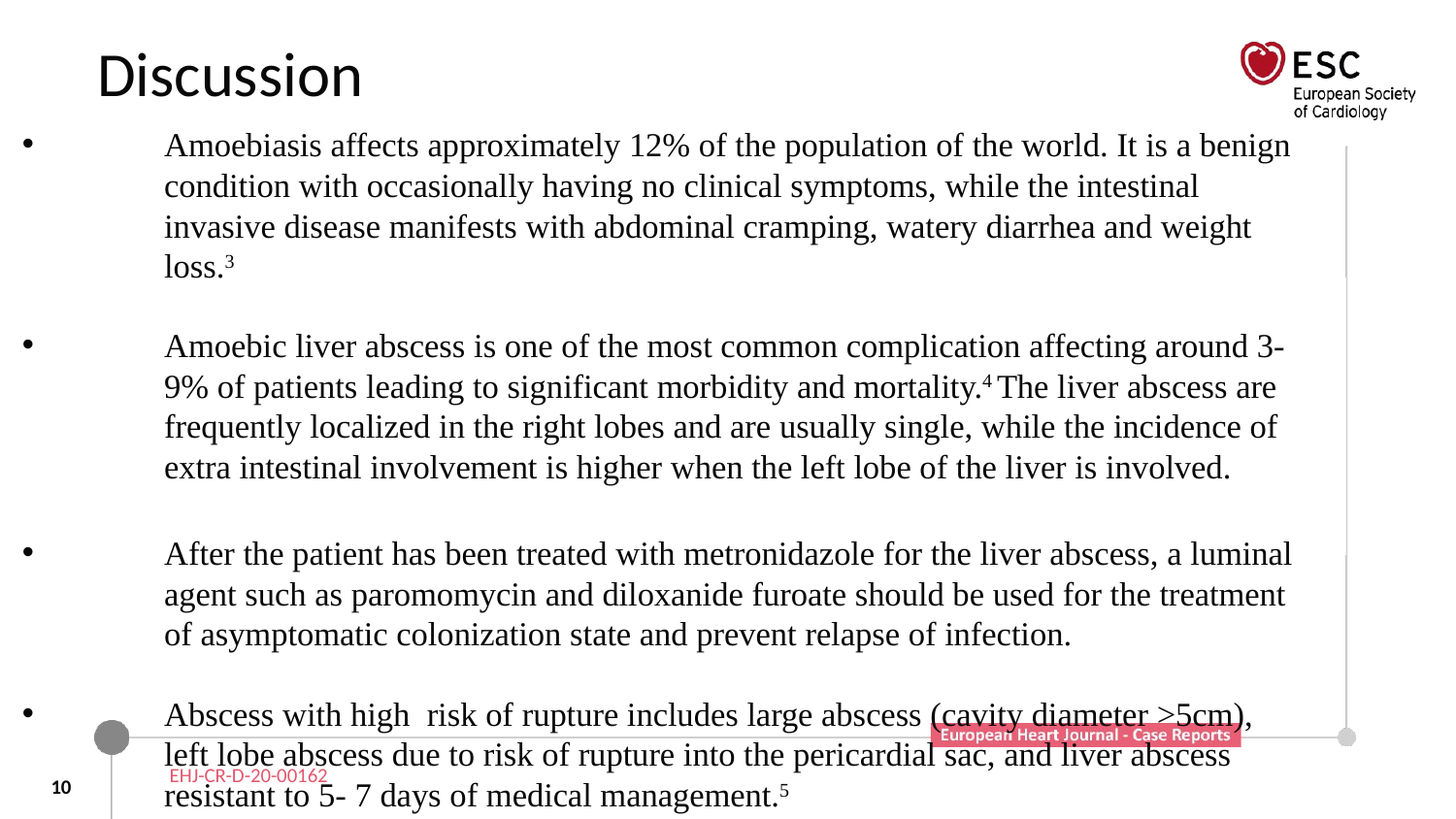

# Discussion
Amoebiasis affects approximately 12% of the population of the world. It is a benign condition with occasionally having no clinical symptoms, while the intestinal invasive disease manifests with abdominal cramping, watery diarrhea and weight loss.3
Amoebic liver abscess is one of the most common complication affecting around 3-9% of patients leading to significant morbidity and mortality.4 The liver abscess are frequently localized in the right lobes and are usually single, while the incidence of extra intestinal involvement is higher when the left lobe of the liver is involved.
After the patient has been treated with metronidazole for the liver abscess, a luminal agent such as paromomycin and diloxanide furoate should be used for the treatment of asymptomatic colonization state and prevent relapse of infection.
Abscess with high risk of rupture includes large abscess (cavity diameter >5cm), left lobe abscess due to risk of rupture into the pericardial sac, and liver abscess resistant to 5- 7 days of medical management.5
10
EHJ-CR-D-20-00162

## Slide 11
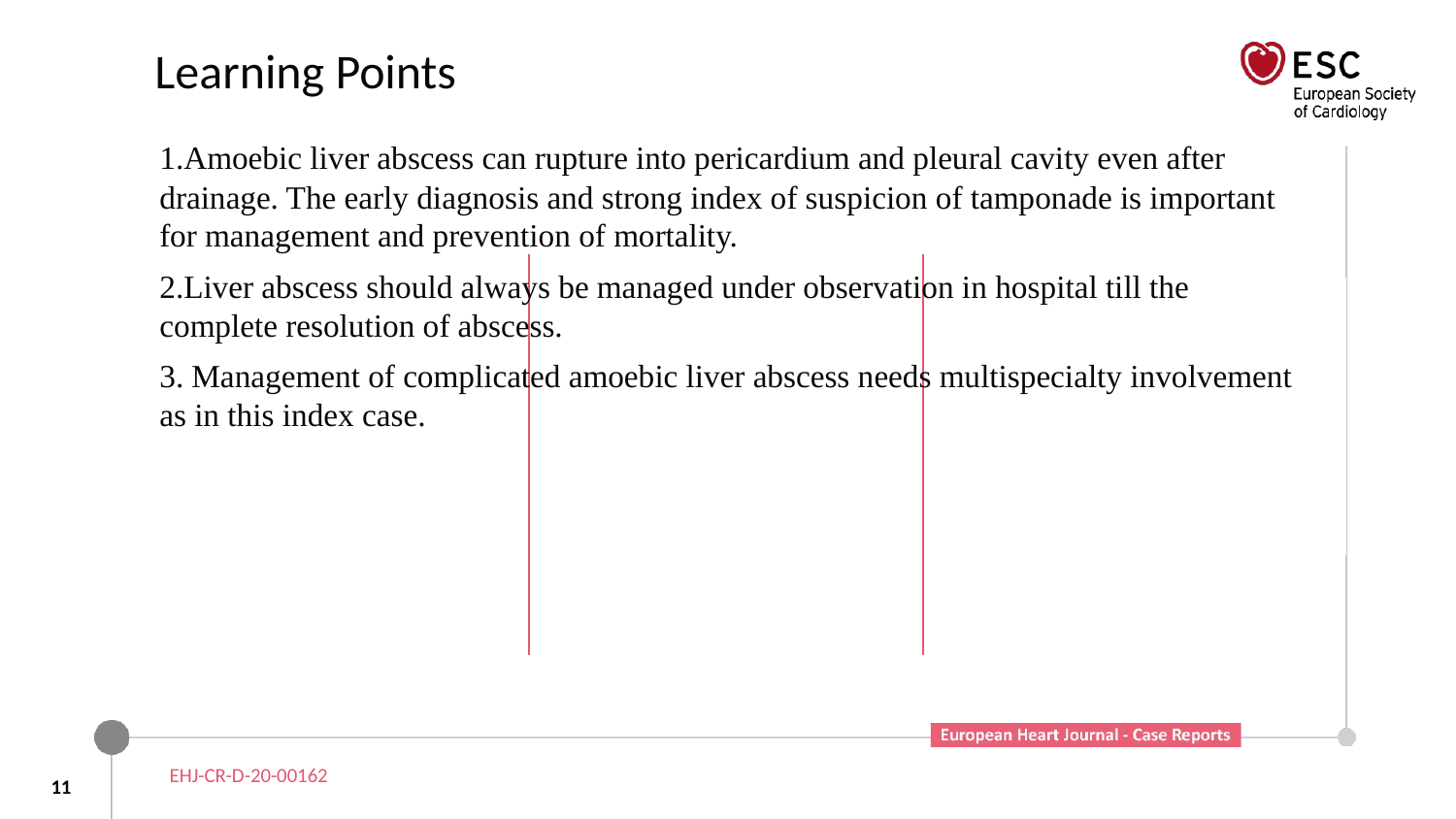

# Learning Points
1.Amoebic liver abscess can rupture into pericardium and pleural cavity even after drainage. The early diagnosis and strong index of suspicion of tamponade is important for management and prevention of mortality.
2.Liver abscess should always be managed under observation in hospital till the complete resolution of abscess.
3. Management of complicated amoebic liver abscess needs multispecialty involvement as in this index case.
11
EHJ-CR-D-20-00162

## Slide 12
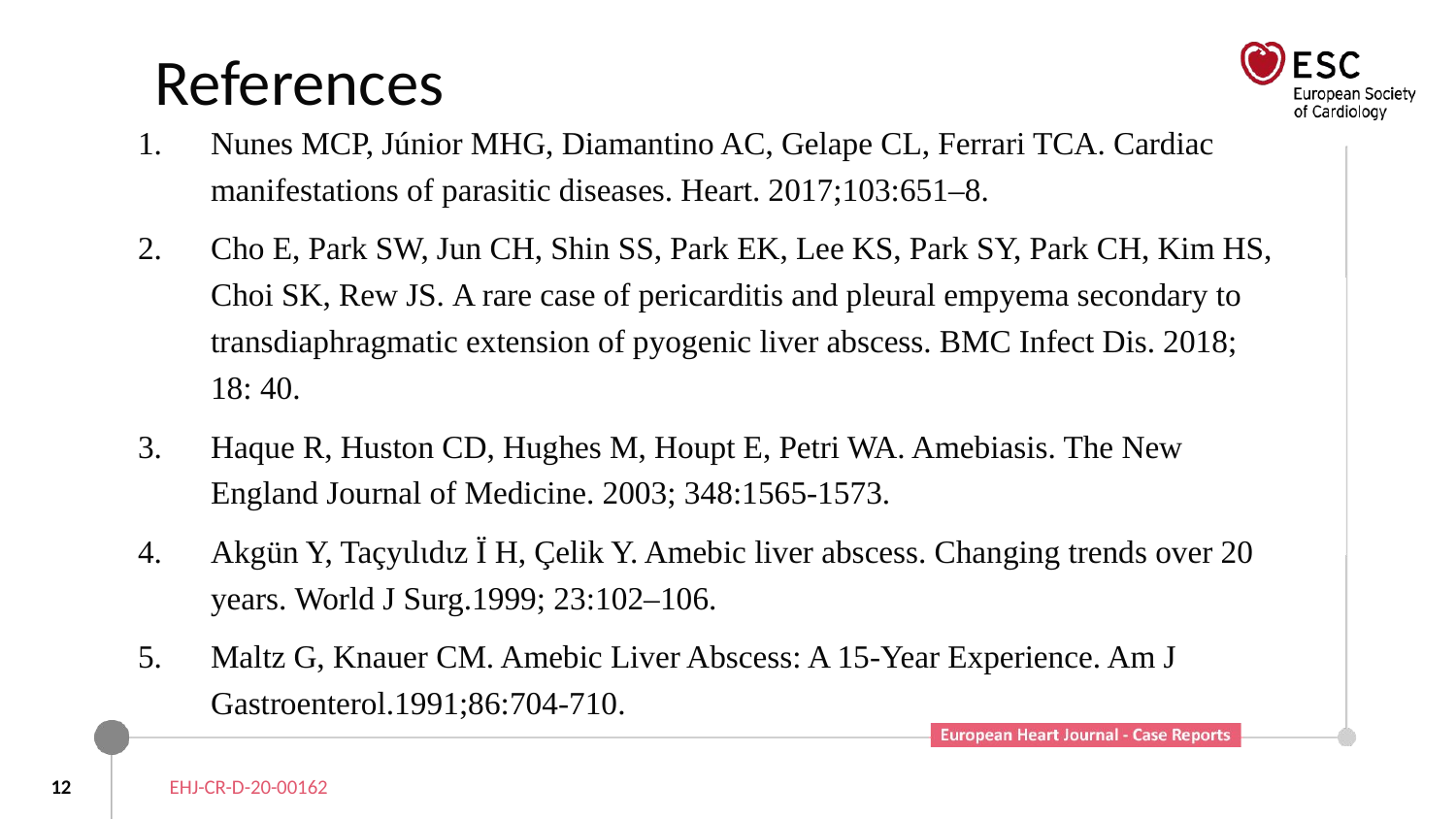

# References
Nunes MCP, Júnior MHG, Diamantino AC, Gelape CL, Ferrari TCA. Cardiac manifestations of parasitic diseases. Heart. 2017;103:651–8.
Cho E, Park SW, Jun CH, Shin SS, Park EK, Lee KS, Park SY, Park CH, Kim HS, Choi SK, Rew JS. A rare case of pericarditis and pleural empyema secondary to transdiaphragmatic extension of pyogenic liver abscess. BMC Infect Dis. 2018; 18: 40.
Haque R, Huston CD, Hughes M, Houpt E, Petri WA. Amebiasis. The New England Journal of Medicine. 2003; 348:1565-1573.
Akgün Y, Taçyιlιdιz Ï H, Çelik Y. Amebic liver abscess. Changing trends over 20 years. World J Surg.1999; 23:102–106.
Maltz G, Knauer CM. Amebic Liver Abscess: A 15-Year Experience. Am J Gastroenterol.1991;86:704-710.
12
EHJ-CR-D-20-00162
